# Efavirenz’ adverse reactions: a possible link between depression and gynecomastia

**DOI:** 10.1186/1471-2334-14-S7-P27

**Published:** 2014-10-15

**Authors:** Ioan Alexandru Diaconu, Daniela Adriana Ion, Victoria Aramă, Adriana Hristea, Ruxandra Moroti

**Affiliations:** 1 National Institute for Infectious Diseases "Prof. Dr. Matei Balş", Bucharest, Romania; 2 Carol Davila University of Medicine and Pharmacy, Bucharest, Romania

## Background

Reports show that 1.8–8.4% of male patients receiving efavirenz (EFV) develop gynecomastia by unclear multiple mechanisms, one of them being a direct binding to estrogen receptors. However, EFV can independently cause gynecomastia by increasing prolactin levels and we postulate that this mechanism is a dopamine-mediated one. Dopamine is a strong prolactin inhibitor. Conversely, a lack of dopamine can cause hyperprolactinemia. On the other hand, dopamine is an antidepressant neurotransmitter and depression is one of the very frequent adverse reactions to EFV. We present the case of an HIV-positive newly diagnosed male patient who developed mild depression and amplified a preexistent gynecomastia after the introduction of an EFV-containing antiretroviral regimen.

## Case report

A 41-year-old male patient, diagnosed with CDC-A2 HIV infection, had been receiving antiretroviral treatment (ART) for 14 months with ABC+3TC+EFV and had been favorably evaluated regarding the immuno-virological course. Nevertheless, after 6 months of ART he developed bilateral gynecomastia with mastodynia and he reported also mild depression. A careful anamnesis discovered a previous mild gynecomastia with also mild rising in prolactin level. Current clinical examination showed moderate bilateral concentric gynecomastia. The breast echography was normal. Brain MRI was performed and can’t exclude microprolactinoma. Lab findings showed normal levels of estrogen and testosterone and high levels of prolactin. We considered dropping EVF and replacing it with raltegravir. After switching (raltegravir doesn’t affect the endocrine ax) mastodynia ceased in one month, the breast volume regressed in 2-3 months and the prolactin level decreased. Besides, the depression’s symptoms diminished.

## Conclusion

In the presented case, gynecomastia was related to hyperprolactinemia and the last was related to EFV use. Moreover, the patient presented also an EFV related depression. It makes sense to consider a unique mechanism for both EFV’s adverse reactions: a drop in dopamine-level caused by EFV, which produce depression and also a hyperprolactinemia with gynecomastia.

